# An Integrative Analysis of The Micro-RNAs Contributing in Stemness, Metastasis and B-Raf Pathways in Malignant Melanoma and Melanoma Stem Cell

**DOI:** 10.22074/cellj.2021.7311

**Published:** 2021-07-17

**Authors:** Parisa Sahranavardfard, Zahra Madjd, Amirnader Emami Razavi, Alireza Ghanadan, Javad Firouzi, Pardis Khosravani, Saeid Ghavami, Esmaeil Ebrahimie, Marzieh Ebrahimi

**Affiliations:** 1. Department of Stem Cells and Developmental Biology, Cell Science Research Center, Royan Institute for Stem Cell Biology and Technology, ACECR, Tehran, Iran; 2. Department of Pathology, Iran University of Medical Sciences, Tehran, Iran; 3. Iran National Tumor Bank, Cancer Institute of Iran, Tehran University of Medical Sciences, Tehran, Iran; 4. Department of Dermatopathology, Razi Skin Hospital, Tehran University of Medical Sciences, Tehran, Iran; 5. Department of Human Anatomy and Cell Sciences, University of Manitoba, Manitoba, Canada; 6. Biology of Breathing, Children Hospital Research Institute of Manitoba, University of Manitoba, Winnipeg, Canada; 7. Autophagy Research Center, Shiraz University of Medical Sciences, Shiraz, Iran; 8. Research Institute in Oncology and Hematology, Cancer Care Manitoba, University of Manitoba, Winnipeg, Canada; 9. School of Animal and Veterinary Sciences, The University of Adelaide, Adelaide, Australia; 10. Genomics Research Platform, School of Life Sciences, College of Science, Health and Engineering, La Trobe University, Melbourne, Australia

**Keywords:** Epithelial-Mesenchymal Transition, Melanoma, MicroRNA, Network Analysis

## Abstract

**Objective:**

Epithelial-mesenchymal transition (EMT) and the stemness potency in association with *BRAF* mutation are in dispensable to the progression of melanoma. Recently, microRNAs (miRNAs) have been introduced as the regulator of a multitude of oncogenic functions in most of tumors. Therefore identifying and interpreting the expression patterns of these miRNAs is essential. The present study sought to find common miRNAs regulating all three important pathways in melanoma development.

**Materials and Methods:**

In this experimental study, 18 miRNAs that importantly contribute to EMT and have a role in regulating self-renewal and the BRAF pathway were selected based on current literature and cross-analysis with available databases. Subsequently, their expression patterns were evaluated in 20 melanoma patients, normal tissues, serum from patients and control subjects, and melanospheres. Pattern discovery and integrative regulatory network analysis were used to find the most important miRNAs in melanoma progression.

**Results:**

Among 18 selected miRNAs, miR-205, -141, -203, -15b, and -9 were differentially expressed in tumor samples than normal tissues. Among them, miR-205, -15b, and -9 significantly expressed in serum samples and healthy donors. Attribute Weighting and decision trees (DT) analysis presented evidence that the combination of miR-205, -203, -9, and -15b can regulate self-renewal and EMT process, by affecting *CDH1, CCND1,* and *VEGF* expression.

**Conclusion:**

We suggested here that miR-205, -15b, -203, -9 pattern as the key miRNAs linked to melanoma status, the pluripotency, proliferation, and motility of malignant cells. However, further investigations are required to find the mechanisms underlying the combinatory effects of the above mentioned miRNAs.

## Introduction

Epithelial to mesenchymal transition (EMT) is that major mechanism involved in increasing the mortality and morbidity of malignancies including melanoma (1). The induction of EMT requires key transcription factors, including snail family transcriptional repressor (SNAIL), zinc finger E-box binding homeobox (ZEB), and twist family bHLH transcription factor (TWIST), that promote epithelial cell reprogramming to repress expression of adhesion molecules, particularly E-cadherin, to initiate migration and invasion (2). The association between the EMT process and stem cell properties in cancer cells has been reported in various tumor cells (3). Studies on malignant tumors, such as melanoma, have reported the involvement of cancer stem cells (CSCs) in tumor initiation, drug resistance, metastasis, and their possible role in cancer recurrence (4). Moreover, based on genomewide analyses, the mutation in the 600^th^ codon of the *BRAF* gene which is the substitution of glutamic acid for valine, is present in about 52% of patients with melanoma and in nearly 15% of all human cancers (5). BRAF encodes a protein belonging to the mitogen-activated protein kinases (MAPK) pathway (6), which mediates a significant role in the regulation of cell division, cell differentiation, and drug resistance (7). On the other hand, through cross talk with the PI3K signaling pathway, the oncogenic BRAF induces EMT and facilitates cell invasion and metastasis (8) and contributing in self rewal potency of melanoma stem cells (9). Therefore these three key pathways; EMT, stemness and *BRAF* play important role in melanoma progression and targeting them has been proposed as the main strategy for successful treatment of melanoma (10). 

miRNAs are an evolutionarily conserved group of small regulatory noncoding RNAs with an approximate length of 22 nucleotides (11). They regulate gene expression by promoting target degradation or translational repression not only during normal development, but also under condition of various diseases, such as cancers (12). Each miRNA can regulate several mRNAs expression. Therefore, miRNAs play key roles in the development of several cancer-related hallmarks (13) and could be considered as prognostic and diagnostic marker, tumorigenicity inducer, migration and even invasion regulator (14), in most of cancers including melanoma.

Based on various investigations on the regulatory role of miRNAs in melanoma, this study was designed to find miRNAs that can simultaneously target multiple processes, which involved in melanoma progression including EMT, stemness, and BRAF pathway. To this end, we used a combination of experimental and computational methods to illustrate the miRNAs and their effect in regulating melanoma progression.

### Materials and Methods

#### Clinical specimens and human ethics

The present experimental study was conducted with the approval of the Ethical Committee of the Royan Institute (code: IR ACECR ROYAN REC.1394.111). In order to perform this experimental study, melanoma specimens were sampled from January 2007 to May 2014 upon the approval of the Iranian National Tumor Bank (INTB) of the Cancer Institute of Iran, obtained based on INTB regulations. The Ethics Committee of INTB had obtained patients’ approval according to local authorities. All contributors signed a written form of consent to enroll in this study. Patients histopathological information, including tumor size and depth, lymph-vascular and perineural invasion, grade and the clinical tumor/node/ metastasis was recorded and pathologically staged using the tumor-nodes metastasis (TNM) staging method (15). All specimens were frozen within 20 minutes after surgery, using nitrogen vapor based on Tumor Bank standard operating protocols.

Twenty patients with malignant melanoma who underwent surgery at the Cancer Institute of Iran were malignancies were excluded from the study. Normal adjacent biopsies were collected from included in this research. The malignant melanoma was confirmed based on histopathological examination in patients. None of the patients had been treated with radio- or chemotherapy prior to surgery. Subjects with chronic or acute inflammatory diseases, other skin cancers, and/or any other all twenty patients as negative controls. In addition, serum samples were taken from 11 patients and 5 healthy donors.

#### Culture conditions and melanosphere formation

Three human melanoma cell lines (A375, D10, and NA8) with BRAF V600E mutation were kindly provided by Prof. Giulio Spagoli (University of Basel, Switzerland). Cells were cultured in Dulbecco’s Modified Eagle’s Medium (DMEM) supplemented with 10% fetal bovine serum (FBS), 1% non-essential amino acids (NEAA), 2 mM L-glutamine, and 1% penicillin/streptomycin (Gibco, Germany). Cell culture was performed in an incubator operating at 37˚C and 5% CO_2_ . 

The formation of melanospheres was established based on a previously published protocol (16). Briefly, 10^4^ cells/ml were grown in six-well plates coated with 12 mg/ml poly 2-hydroxyethyl methacrylate (Sigma, Germany). Serum-free DMEM containing 1% NEAA, 2 mM L-glutamine, 1% penicillin/streptomycin, 1x B-27 supplement (Gibco, Germany), 20 µg/ml epidermal growth factor (EGF, Royan, Iran), and 20 µg/ml basic fibroblast growth factor (bFGF, Royan, Iran) was used for culturing melanospheres. Every 48 hours, fresh B27, bFGF, and EGF were added to the culture medium. Melanospheres were passaged three times in total, once every seven days.

#### MiRNA selection based on literature and database mining

To identify possible miRNAs associated with EMT in melanoma, we first performed a systematic search on PubMed and Scopus using "microRNA" and "melanoma"as keywords in the title of papers published between 2007 and 2016. Then we excluded manuscripts if there were no correlations with "epithelial-mesenchymal transition", "metastasis", and "invasion". Parallel database mining was performed by using the Kyoto Encyclopedia of Genes and Genomes (KEGG) Pathway Database to find genes associated with EMT signaling, the BRAF pathway, and stemness. Subsequently, further analysis was conducted using miRNA databases, which were available in miRTarBase (http://mirtarbase.mbc.nctu.edu.tw/), TargetScanHuman (www.targetscan.org/), and miRCancer (http://mircancer.ecu.edu/) (17-19), to find miRNAs that directly regulate these genes. Finally, potent miRNAs in the regulation of self-renewal, invasion, migration, and metastasis in malignant melanoma were selected by cross-analysis of the results of literature and database mining.

#### Quantification of miRNAs and mRNA by real-time quantitative reverse transcription polymerase chain reaction

Trizol® Reagent (Invitrogen, USA) was used to extract total RNA from melanoma cells, melanospheres, and tissues. Total RNA extraction from serum was performed using miRNAeasy kit (Qiagen, USA). All procedures were conducted according to the manufacturer’s instructions. Reverse transcription of 2 μg of miRNAs and mRNAs was carried out using MiR-Amp kit (PARSGENOME, Iran) and Thermoscript (TaKaRa, China), respectively. Next, real-time quantitative reverse transcription polymerase chain reaction (qRT-PCR) using Power SYBR® Green (Applied Biosystems®, UK) was applied to quantify the expression levels of miRNAs and mRNAs in duplicate (7500 Fast qRT-PCR System, Applied Biosystems, CA). The qRT-PCR was performed in three step: 30 seconds at 95˚C as hold time, 40 cycles of denaturation at 95˚C for 5 seconds, annealing at 60˚C for 20 seconds, and 30 seconds extension at 72˚C. Melting curves were determined from 55 to 99˚C. The expression level of each miRNA was normalized against U6 snRNA expression and *GAPDH* was used to normalize mRNAs. The quantitative 2^-ΔCt^ method was adopted for calculating the individual expression levels of patients’ miRNAs in tumor and normal samples. The relative quantitative approach (2^-ΔΔCt^) was used to demonstrate relative expression of target genes of miRNAs, and miRNAs and mRNAs levels in melanospheres. GraphPad Prism 6 was used for data analysis and graph preparation.

### Univariate statistical analysis

Categorical variables were assessed using proportion tests including Z-test and Fisher’s exact test. t tests were applied to compare numerical data (presented as mean ± standard error of the mean). The statistical comparisons were performed using R software (version 3.0.2), Minitab17, and GraphPad Prism version 7 (San Diego, USA). 

### MiRNA pattern recognition based on data mining

In an attempt to i. Identify the major miRNAs distinguishing between tumor and normal samples, ii. Determine the combination and hierarchy of miRNAs which had the highest accuracy in predicting tumor development, and iii. Calculate the predictive power of the created model using cross-validation, a comprehensive data mining analysis was applied. For this purpose, 10 different attribute weighting models and 176 combinational decision tree (DT) models were developed.

### Attribute weighting

Ten different attribute weighting algorithms were applied to determine the main miRNAs that could accurately discriminate between melanoma and normal samples (Table S1, See Supplementary Online Information at www.celljournal.org). Following attribute weighting, the weights were normalized and miRNA attributes received a value between 0 and 1. Values, which were closer to 1, showed higher importance of that particular miRNA in the discrimination between normal and tumor samples according to the employed models. Variables weighted as ≥0.9 were then selected and with tree induction algorithms were used to predict the cancer development.

### Decision tree and random forest models

As the most popular supervised learning methods for data exploration, DT classifiers facilitate easy interpretation by summarizing and transforming data into more compact forms with the same essential characteristics as the original data. As described earlier, 10-fold cross-validation was adopted to identify the DT models most accurately predicting cancer development. 

### Enrichment analysis for signaling pathways using fisher’s exact test

Enrichment analysis was employed to find the significant regulatory mechanisms of differentially expressed miRNAs using Pathway Studio Web tool (18). 

The statistical significance (P values) of enriched annotation terms was determined using Fisher’s exact test. Lower P values indicated greater enrichment, P≤0.05 were considered significant.

### Interaction network database

We used the Mammalian+ChemEffect+DiseaseFx Database (Elsevier), which is a comprehensive dataset of proteins, small molecules, diseases, Gene Ontology, and functions collected by a natural language processing (NLP) tool (19). The relations were collected from PubMed, KEGG, Science Signaling, GO Consortium, and Prolexys HyNet protein-protein interaction databases as well as full texts of relevant papers in both Elsevier and non-Elsevier journals. The database contains 284400 entities, 7151512 relationships, and 2023 pathways. Pathway Studio was used to build networks and pathways from relationships of Mammalian+ChemEffect+DiseaseFx Database.

### Common targets common regulators algorithms

Pathway Studio Web tool (19) was used for 'common targets' and 'common regulators' analysis. A component (gene /miRNA) is regarded as a common regulator when it has a high number of upstream interactions with the differentially expressed miRNAs. We optimized this parameter and set a threshold of three interactions. Likewise, a component is considered as a common target if it has a high number of downstream interactions with the differentially expressed miRNAs. After the evaluation of various values, a threshold of three interactions was set for the analysis.

Differentially expressed miRNAs were used as the input of common targets and common regulators algorithms. The common targets algorithm identified the targets/mechanisms, which were activated/ inactivated by the altered miRNAs, i.e. it sought to clarify the goal/ consequence of the determined miRNAs modulation pattern. However, the common regulators algorithm determined the regulators with the maximum number of regulation/expression relationships with the altered miRNAs, i.e. it sought to identify the managers/ commanders/regulators of the altered miRNAs.

### Survival analysis and definition of miRNA-related prognostic signature

For assessment of overall survival implications for significant miRNAs, the PROGmiR tool (20) was used as a publicly available dataset (http://www.compbio.iupui. edu/progmir). The melanoma expression data comes from the TCGA dataset (https://cancergenome.nih.gov), including 163 cases of skin cutaneous melanoma.

### Construction of the tissue microarray 

A total of 12 archival tissue samples of melanoma (Shohada-e-Tajrish Hospital, Iran) were used for tissue microarray analysis (TMA). Medical records were reviewed to collect the clinicopathological data (Table S2, See Supplementary Online Information at www. celljournal.org). The study protocol was approved by the Research Ethics Committee of Iran University of Medical Sciences.

For TMA, 12 melanoma and 7 adjacent normal tissues of hematoxylin and eosin-stained slides were reviewed to determine the best pathological area from each specimen. The slides were then prepared by placing duplicate samples (0.6 mm in diameter) from each specimen using a manual tissue-arraying instrument (Minicore; ALPHELYS, Plaisir, France). These slides were used for immunohistochemical staining. 

### Immunohistochemistry

The expression of CDH1 and SOX2 were immunohistochemically evaluated using the manufacturer’s protocol. After initial preparation, the sections were incubated overnight at 4˚C with rabbit polyclonal E-cadherin antibody recognizing the extracellular domain of E-cadherin (1:300 dilution, H-108, Santa Cruz Biotechnology, USA), and specific antibody against rabbit monoclonal anti-human SOX2 (1:250 dilution, cat. 3579, Cell Signaling, USA). The sections were washed the next day and incubated with the anti-rabbit/anti-mouse EnVision reagent (Dako, Denmark), as the secondary antibody, for 60 minutes. The sections were then stained with 3, 3’-diaminobenzidine (DAB, Dako) substrate as chromogen for two minutes in the dark and at room temperature. Subsequently, the sections were counterstained with hematoxylin (Dako, Denmark), dehydrated through graded ethanol followed by xylene, and mounted. Normal human brain tissue and ovarian carcinoma were used as positive control for SOX2 and E-cadherin antibodies respectively. The negative control was incubated only with Tris-buffered saline (TBS).

### Immunohistochemical evaluation and scoring

Two independent observers used a multi-headed microscope to evaluate the stained slides based on a semi-quantitative scoring system. The scoring was executed without previous knowledge of clinicopathological data. The intensity of staining was scored as 1+ (weak), 2+ (moderate), or 3+ (intense) and the percentage of positive tumor cells was scored as 1 (positive tumor cells<25%), 2 (positive tumor cells: 25-50%), 3 (positive tumor cells: 50- 75%), and 4 (positive tumor cells >75%). The histochemical score (H-score) was ultimately calculated as the product of staining intensity and the percentage of positive tumor cells by multiplying the intensity of staining and the percentage of positive tumor cells.

## Results

### Patient demography

Specimens obtained from 20 patients with cutaneous malignant melanoma were evaluated in this study.

Patients’ age varied between 38 and 83 years, with 60% of the subjects being older than 65 years. In nine patients (45%), the primary tumor site was at the lower limb and hip and 50% of all patients had ulcerations. According to the TNM classification of malignant melanoma, 65% of patients had stage II melanoma (Tables S3, S4, See Supplementary Online Information at www.celljournal.org).

### miRNA slection

**Fig.1:**
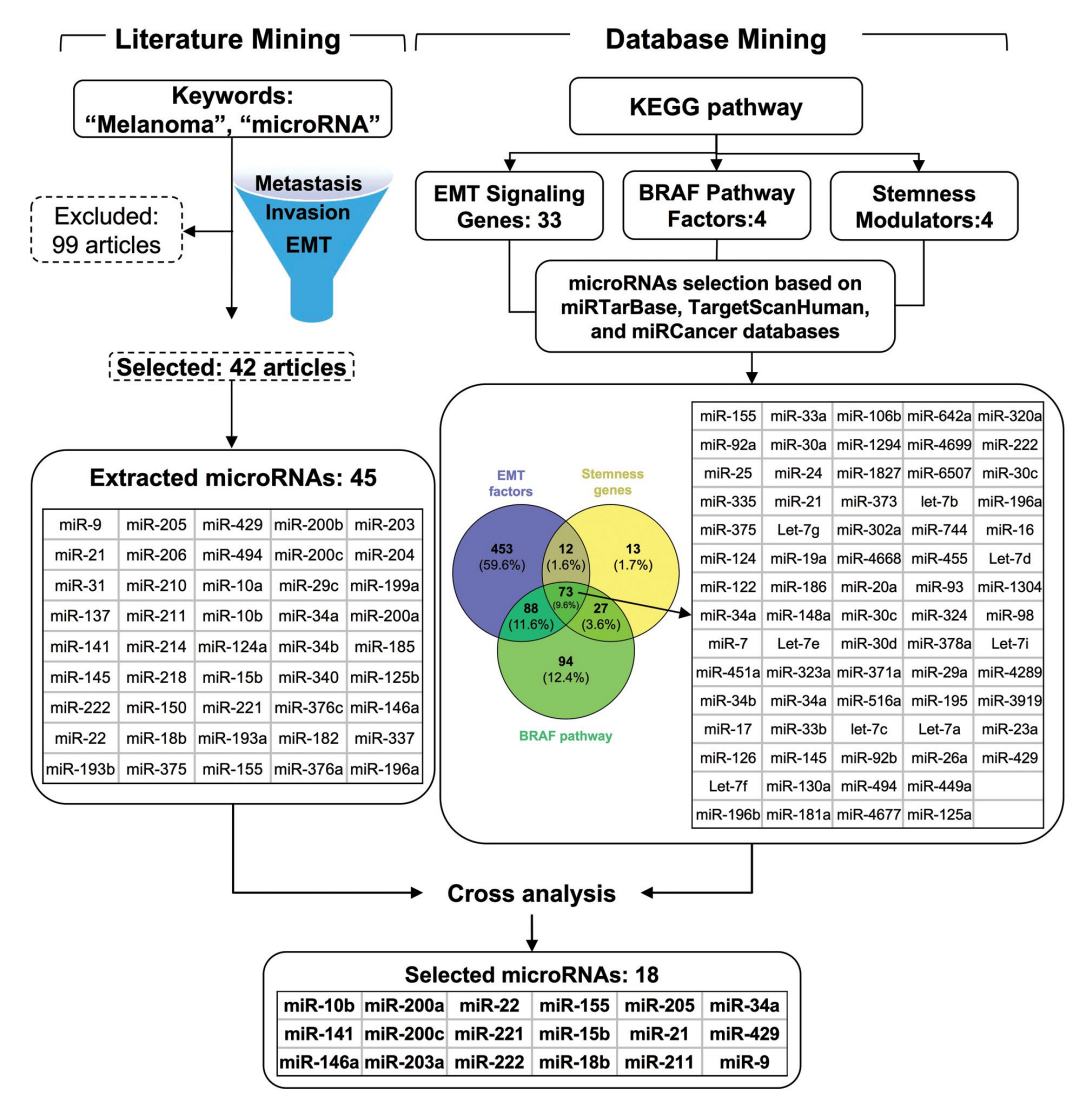
Schematic illustration of miRNA selection procedure. MiRNAs were selected using literature and database mining. Ultimately, 18 miRNAs were selected following cross-analysis.

To gain further insight into miRNAs that simultaneously control EMT, stemness, and the BRAF pathway, literature mining and cross-analysis with available databases were performed, as described in the Methods section. Literature mining resulted in 141 articles that were published between 2007 and 2016, and contained the predetermined keywords "microRNA" and "melanoma" in the title. Of those, 99 articles were excluded as they did not meet our selection criteria (correlation to EMT, metastasis, invasion and stemness features) and also because of data duplication. Finally, 45 miRNAs were selected from 42 articles. Parallel database search resulted in a total of 626 miRNAs (including 33 target genes) contributing to the EMT process. Interestingly, 85 and 161 of these also targeted stemness modulators (including four target genes) and BRAF pathway factors (including four target genes), respectively. Finally, 73 miRNAs were identified to target all three processes of EMT, stemness, and metastasis. However, only 18 miRNAs (miR-9, 10b-15b, 18b-21, 22-34a, 141-146a, 155-200a, 200c-203, 205-211, 221-222, and-429) were ultimately selected following the cross-analysis of the miRNAs extracted from literature and database mining (Fig.1, Fig Supplementary Excel 1, See Supplementary Online Information at www.celljournal.org).

### Differential expression of miRNAs in melanoma, normal adjacent tissue, and serum

**Fig.2:**
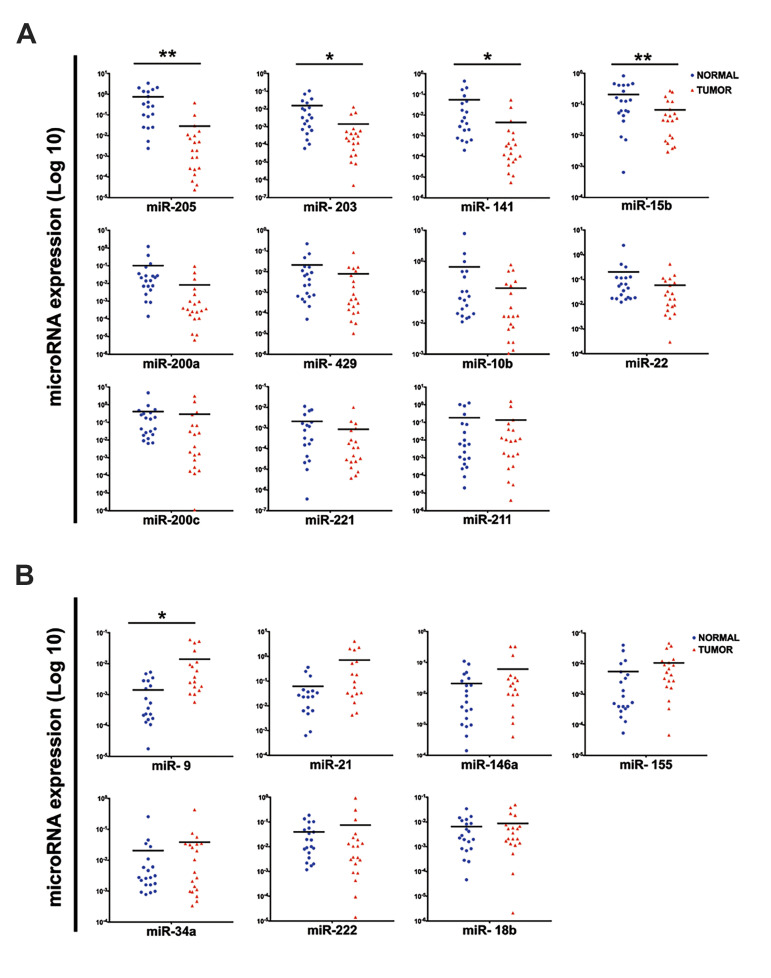
The expression pattern of selected miRNAs in melanoma and normal adjacent tissue. **A.** The significant down regulation of miR-205, -141, -203, -15b was observed in melanoma tissues (n=20, Log 10, *; P<0.05, **; P<0.01) and **B.** Scatter-plots of the expression levels of the selected miRNAs show a significant higher expression of miR-9 (n=20, Log 10, *; P<0.05) in melanoma samples compared with normal adjacent tissues.

Among 18 selected miRNAs, the expression of 5of them (miR-205, 141-203, 15b-and, 9) was significantly different between groups. The expression of miR-205, -203, -141 and -15b was decreased in tumor samples in comparison with normal adjacent tissues, and the expression of miR-9 was significantly higher in tumor samples as compared to the normal group (P<0.05, Fig.2).

**Fig.3:**
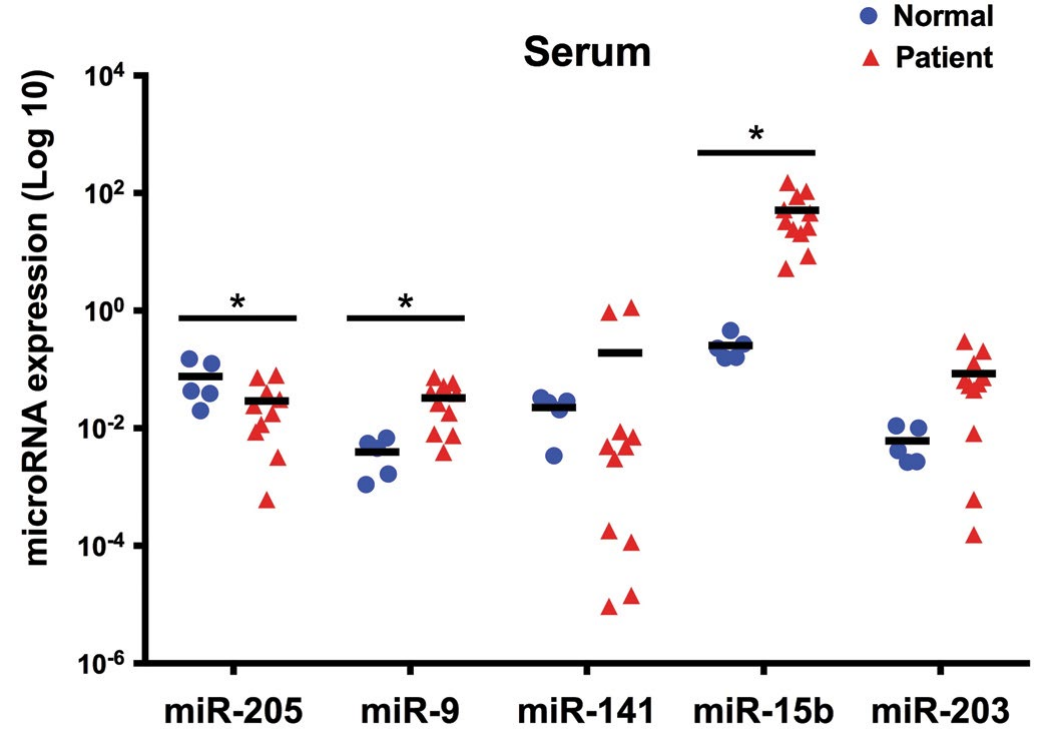
The expression pattern of selected miRNAs in serum obtained from melanoma patients and healthy donors. Among five miRNAs, miR-205, -15b, and -9 showed significant differences (Log 10, *; P<0.05) between serum obtained from melanoma patients (n=11) and control subjects (n=5).

According to validated data (miRTarBase 6.0: Sept. 15, 2015), all of these 5 miRNAs had at least one target in the EMT pathway: miR-205 and miR-141 target *ZEB*; miR-203 targets *ZEB, SNAIL,* and *SMAD2*; miR-15b targets *SMAD2*; miR-9 targets *CDH1* and *SNAIL*. Moreover, with regards to stemness genes, miR-141 inhibits *POU5F1* and miR-9 directly targets *SOX2*. In addition, miR-9, -15b, and -203are involved in the BRAF pathway by directly targeting *BRAF* or one of its downstream factors, like *ERK, MEK *or *CCND1* (Fig.S1, See Supplementary Online Information at www.celljournal.org). Comparison of the expression of miR-205, -141, -203, -15b, and -9 in the serum of patients and healthy donors revealed significant differences for miR-205, -15b, and -9 (P<0.05, Fig.3). The expression patterns of miR-205 and miR-9 in serum from patients were similar to those of tumor samples. However, in contrast to tumor samples, serum obtained from patients showed increased expression of miR-15b as compared to serum from control subjects. 

### Predicting the most important melanoma-linked miRNAs and developing predictive models using attribute weighting and decision tree by random forest

MiR-205, -15b, and -141 were selected as the key miRNAs linked to tumor/normal status using four-attribute weighting models with different statistical backgrounds (Table S5, See Supplementary Online Information at www.celljournal.org). MiR-205 received the highest weight of 1 by information gain ratio, information gain, and Gini index. It was also weighted as 0.9 by the uncertainty model (Supplementary Excel 2, See Supplementary Online Information at www.celljournal. org).

In order to identify the best combination of miRNAs that distinguished between healthy and malignant status, extensive computational biology analysis was applied to test DT based on expression of miRNAs. Distinguished capability decision-tree classifier in highly accurate identification of cancer origin based on miRNA profile has been documented (21). Also, DT models have shown high applicability for accurate classification of kidney cancer subtypes using miRNAs signature (22). Therefore, we used DT model for finding the hierarchical combination of miRNAs as a biomarker for melanoma. On the otherhand, 10 attribute weighting algorithms were applied with various statistical backgrounds to determine the main miRNAs that could accurately discriminate between melanoma and normal samples. We selected miRNAs based on the intersection/agreement of different models where miRNA receiving high weights by most of models were announced as important ones. Applying these models increased our confidence about the selected miRNAs. The accuracy of each model was evaluated and presented in Supplementary Excel 3 (See Supplementary Online Information at www.celljournal.org). The highest accuracy was obtained by the Random Forest Gain Ratio and Random Forest Info Gain models, which were able to accurately predict tumor/normal status of 90% of the samples (based on cross-validation).

Following attribute weighting on expression of microRNAs (miRNAs) in normal and tumour, the weights were normalized and miRNA attributes received a value between zero and one. Values closer to one showed higher importance of that particular miRNA in discrimination between normal and tumor samples according to the employed model. Variables weighted as ≥0.9 were then selected. For example, in the following Table S5 (See Supplementary Online Information at www.celljournal. org), miR-205 is selected based on statistics of 4 models including Weight_Info Gain Ratio, Weight_Info Gain, Weight_Uncertainty, and Weight_Gini Index to be important in discriminating tumour from normal sample.

**Fig.4:**
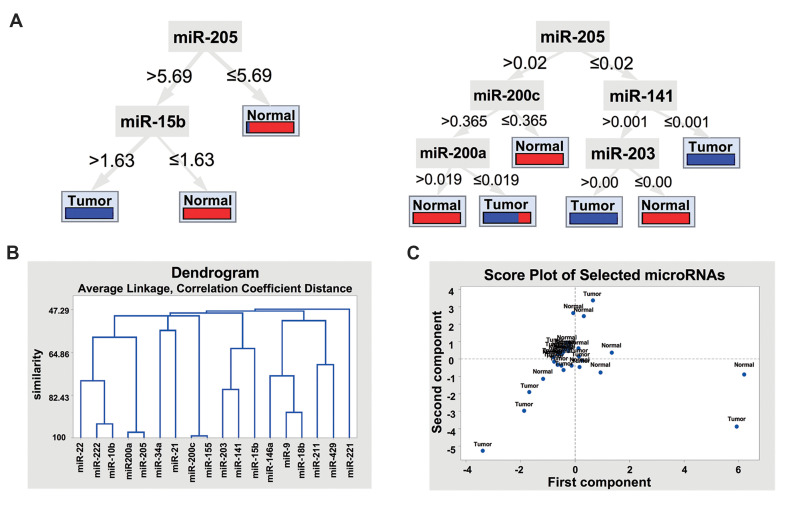
Pattern discovery distinguishing tumor from normal samplesusing machine learning and multivariate analytical models. **A.** Decision tree (DT) model of Random Forest Gain Ratio predicts normal/tumor status based on miRNA expression levels. Random Forest is able to find the threshold in expression of each miRNA. As shown in the results, miR-205 was the key regulator of healthy and malignant status, **B.** Clustering of miRNAs, based on their expression levels, indicates that the expression patterns of miR-205/ miR-200a, miR-200c/ miR-155, and miR-222/ miR-10b in cancer samples were over 95% similar, and **C. **PCA analysis of expression of miRNAs in relation to tumor/normal status exhibited high diversity in tumor samples.

MiR-205 emerged as the key indicator of healthy and malignant status and the combination of high miR-205 expression with lowmiR-200c expression indicated a healthy status. In contrast, low miR-205 and -141 expression was associated with malignancy (Fig.4A, Right panel). Moreover, the low expression of both miR-205 and miR-15b could be indicative of the malignant state (Fig.4A, Left panel). To determine the commonality between miRNAs, they were clustered by hierarchical clustering methods, as previously described (23). Our results revealed that the expression patterns of miR-205, -200c, and -222 in melanoma tissue samples were over 95% similar to those of miR-200a, -155, and -10b, respectively (Fig.4B). As the miRNAs with the similar expression pattern may be regulated by the similar set of transcription factors (common regulators), therefore, we suggested the same transcription factors might regulate these miRNAs. As shown in Figure 4C, tumor samples had high diversity and negative amount of second principal component analysis (PCA).

### Regulatory network in progression to malignant melanoma

A 'regulatory network' sustaining the progress toward malignancy was designed by combining the statistically significant sub-networks of significant miRNAs in Gene Set Enrichment Analysis using Pathway Studio Web tool (Elsevier, Supplementary Excel 4, See Supplementary Online Information at www.celljournal.org). The selected miRNAs were subject to regulation by most intracellular components, including the nucleus, Golgi apparatus, and the cell membrane (Fig.S2A, B, See Supplementary Online Information at www.celljournal. org). The common regulatory factorswere TGFβ1, TP53, and histone deacetylase that regulated six of the seven indicator miRNAs: miR-9, -200a, -200c, -141, -15b and -205 (Fig.S3 ,Fig Supplementary Excel 5, See Supplementary Online Information at www.celljournal.org).

**Fig.5:**
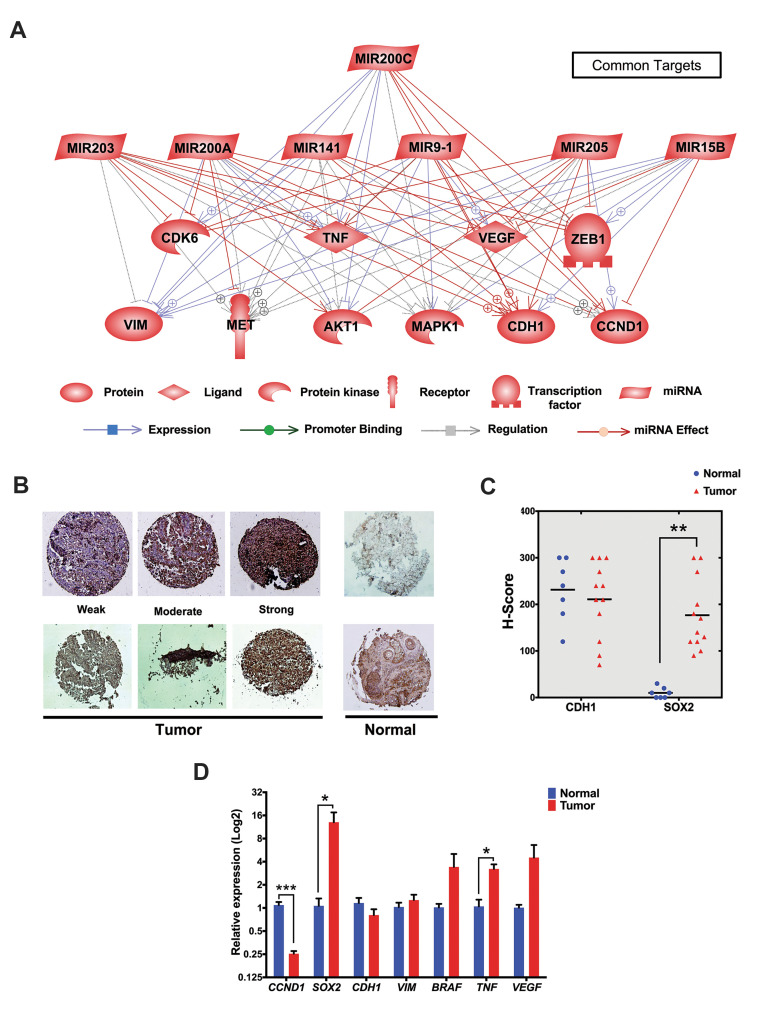
Validation of the most important common regulators and targets of differentially expressed miRNAs. **A.** Analysis of common targets revealed that MET, CDH1, VEGFA, TNF, ZEB, CDH1, and CCND1 represent the key targets of differentially expressed miRNAs, **B, C.** The protein expression levels of SOX2 and CDH1 obtained from Tissue Micro Array data and presented as H-Score. SOX2 expression was elevated in melanoma tissues (n=12) despite of normal skin (n=7), and **D.** mRNA expression levels of *CCND1, SOX2, CDH1, VIM, BRAF, TNF, VEGF. CCND1* was significantly lower expressed in tumor samples, whereas SOX2, BRAF, TNF, and VEGF were overexpressed in malignant tissues compared with normal samples (n≤15, *; P<0.05, **; P<0.01, ***; P<0.001).

Analysis of common targets revealed that MET proto-oncogene (*MET*), *CDH1,* vascular endothelial growth factorA (*VEGFA*), and tumor necrosis factor (*TNF*) were the key targets of these six miRNAs. Also, it seems that miR-200c was the upstream of most important cancer regulators like *ZEB, CDH1, *and *CCND1*as common targets (Fig.5A).

In order to validate the targets, qRT-PCR was performed to assess the mRNA expression of *CDH1, CCND1, SOX2, VIM, BRAF, TNFA *and *VEGF*. According to Figure 5A all of these genes are common targets for miR-205, -203, -9 and -15b. TMA using 12 samples from malignant patients and 7 normal/control tissues showed the higher expression of SOX2 at protein level in melanoma tissues, in comparison with normal skin biopsies. CDH1 protein was highly expressed both in melanoma and normal skin biopsies (P<0.01, Fig.5B, C). Although, at mRNA level, *CCND1* expression was significantly lower in tumor samples (P<0.001, Fig.5D), *SOX2, BRAF, TNFA,* and *VEGF* expression was increased in malignant tissues compared with normal adjacent samples (P<0.05, Fig.5D). 

**Fig.6:**
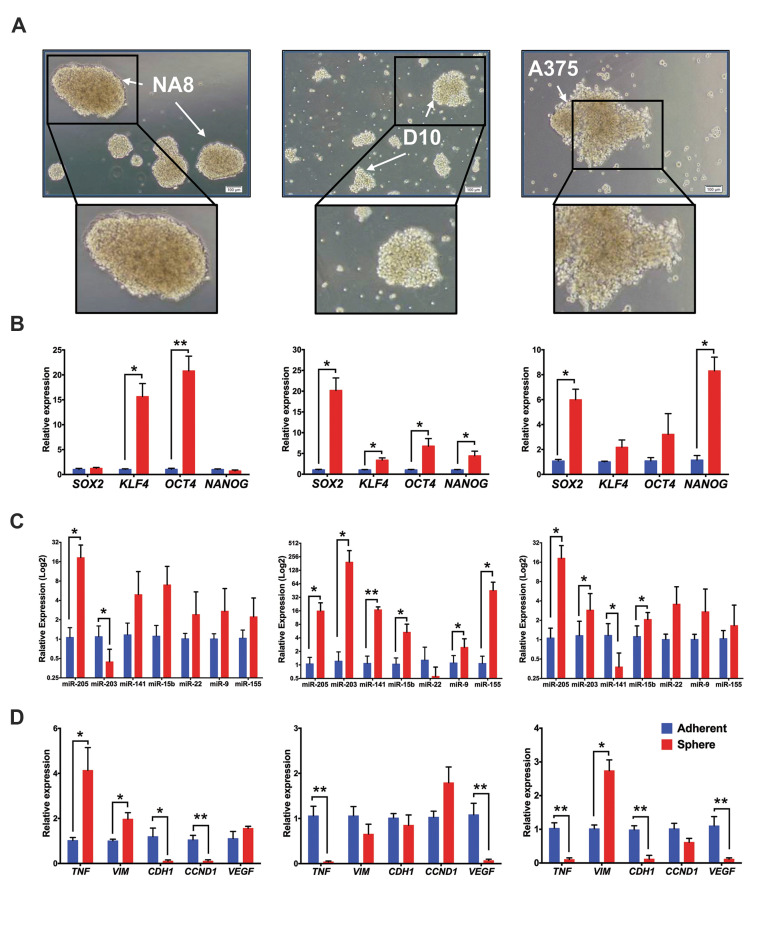
The expression analysis of stemness transcripts, selected miRNAs, and their target genes in melanospheres by quantitative reverse transcription polymerase chain reaction (qRT-PCR). **A. **The morphology of melanospheres derived from NA8 (left), D10 (middle), and A375 (right) revealed that, NA8- melanospheres were compact with defined borders. However, D10 and A375 formed loose, grapelike melanospheres, **B.** Relative expression levels of *SOX2, KLF4, OCT4* and *NANOG* transcripts in NA8-, D10- and A375-melanospheres compared to adherent cells. There were significant upregulations in* KLF4* and *OCT4* expression in NA8-melanospheres. D10 showed upregulation in *SOX2, KLF4, OCT4* and *NANOG* levels and significant upregulation was observed in *SOX2* and *NANOG *in melanosphere derived from A375 cells (n=3, *; P<0.05, **; P<0.01), **C. **The expression of miR-205, -203, -141, -15b, -22, -9, and -155 in melanospheres originating from all three cell lines. The expression of miR-205 was upregulated in all melanospheres compared with parental cells. MiR-203 was significantly upregulated in D10 and A375 melanospheres, unlike in spheres derived from NA8 cells. MiR-15b expression was significantly increased in D10 and A375 melanospheres compared to parental cells. MiR-141 was upregulated in D10 and downregulated in A375. Although miR-9 showed significantly higher expression in D10 melanospheres in comparison with parental cells (n=3, log 2, *; P<0.05, **; P<0.01), and **D.** Relative expression of* CDH1, VIM, TNFA, VEGF,* and *CCND1* transcript in three cell lines. TNFA was significantly downregulated in D10- and A375- and upregulated in NA8-melanospheres compared to parental cells. *VIM* shows higher and *CDH1 *lower expression in NA8- and A375-melanospheres. *CCND1* was significantly downregulated only in NA8- melanospheres. Expression of VEGF was markedly lower in D10 and A375 spheres (n=3, *; P<0.05, **; P<0.01).

### The combined expression of miR-203, -205, -15b, and -9 is associated with survival rates

Based on the PROGmiR database, the individual expression levels of miR-205, -15b, or -9 alone did not significantly correlate with the survival rates of melanoma patients. In contrast, higher expression of miR-203 was significantly associated with reduced survival rate (P<0.05, Fig.S4A, See Supplementary Online Information at www.celljournal.org). On the other hand, the combined expression of them, appears to severely effect overall survival (P=0.0192) in melanoma patients (Fig.S4B, See Supplementary Online Information at www.celljournal. org).

### EMT-miRNAs expression pattern in melanospheres as a cancer stem cell model

To determine if the six selected miRNAs were expressed in melanospheres (melanoma stem like cells), we assessed the expression pattern of those by qRT-PCR. The melanospheres were derived from three different melanoma cell lines. Morphologically, melanospheres derived from NA8 were dense, compact with defined borders; conversely, the D10 and A375 cells formed loose, grapelike melanospheres (Fig.6A). Melanospheres derived from different melanoma cell lines revealed differential expression patterns for stemness genes. Levels of *KLF4* and *OCT4* mRNA were significantly increased in NA8-melanospheres, while D10-melanospheres showed elevated expression of *SOX2, KLF4, OCT4* and *NANOG*, and A375- melanospheres displayed enhanced expression of *SOX2* and *NANOG *(P<0.05, P<0.01, Fig.6B). In all melanospheres, overall the expression levels of miR-205, -203 and -9 were higher than in their parental cells; of note, the expression of miR-203 was significantly decreased in NA8-mellanospheres and miR-9 just significantly expressed in D10-melanosphere (P<0.05, Fig.6C). MiR-15b expression was significantly increased in D10 and A375 melanospheres, whereas miR-141 was differentially expressed, upregulated in D10 (P<0.01, Fig.6C) and downregulated in A375 (P<0.05, Fig.6C). Among the main common targets, *TNF* expression was higher in NA8-melanospheres; conversely, it was reduced inD10- and A375-melanospheres. The level of *VIM* was increased and that of *CDH1*reduced in melanospheres derived from NA8 and A375. NA8-melanospheres had lower levels of *CCND1* expression. Lastly, the expression pattern of *VEGF* was reduced in D10- and A375- melanospheres in comparison with parental cells (P<0.05, P<0.01, Fig.6D).

## Discussion

Alterations in the EMT process and BRAF signalling pathway play a key role in melanoma progression (24, 25), and affect stemness properties involved in metastatic competence and tumor regrowth (26). Nevertheless, further research is required to determine exactly which factors can simultaneously regulate these processes. For this, a systematic analysis based on literature and databases’ mining was performed, and 18 miRNAs were identified. However, the expression of miR-205, -141, -203, -15b, and -9, was significantly different between malignant melanoma and adjacent normal tissues. Expression levels of miR-205, -141, -203, and -15b were lower, whereas expression of miR-9 was higher in melanoma tissue. Interestingly, miR-9, -15b, and -203 are documented to contributeto BRAF pathways by direct targeting of *RAF, MEK,* and *ERK.* Moreover, miR-9 and -141 are associated with stemness properties by targeting *SOX2* and *OCT4.*

In order to distinguish a unique expression pattern of miRNAs, 10 attribute weighting models and DT models were used. Based on these models, we suggested miR-205, -203,-9, and -15b as common regulators of EMT, self-renewal, and BRAF pathways in melanoma. Therefore, we evaluated expression of these miRNAs in patients’ serum and melanospheres derived from NA8, D10, and A375 cell lines. Interestingly, among them miR-205 had a similar expression pattern (low expression) in tumor biopsies and serum of patients in comparison with normal control groups, but showed an increased expression in all groups of cell line melanospheres. Machine learning analysis revealed the reduction of miR-205 level as a key regulator of the malignant state in melanoma, which is in accordance with previous reports in melanoma (27) and gastric cancer (28). Although, its increased expression in melanoma stem cells is still ambiguous, it had positive correlation with OCT4 in all types of melanospheres and with NANOG in melanospheres derived from D10. Therefore, it may be connected to the pluripotent state of melanoma cells. Similar to our results, miR-205 has been reported to be associated with the EMT process, stemness traits of cancer stem cell (CSC) fate, tumorigenicity, and chemoresistance in breast cancer (29) and non-small cell lung cancer (30). The elevated expression of miR-205 in mouse mammary epithelial stem-like cells led to expansion of the progenitor cell population through the suppression of phosphatase and tensin homologue (PTEN) (31). Additionally, the overexpression ofmiR-205 resulted in high proliferation of endometrial and ovarian cancer (32).

MiR-9 displayed a similar expression pattern in patients’ melanoma biopsies, serum, and melanospheres (higher expression as compared to control). According to common regulator analysis, we found that *SOX2* can regulate miR-9 expression, as a SOX2-binding site has been detected in the promoter region of miR-9 (33). Based on our results, enhancement of *SOX2* at the mRNA and protein level in tumor tissues as well as melanospheres, can enhance the expression of miR-9, which results in an increased motility of melanoma cells through reduction of *CDH1* level (34). Moreover, its overexpression increases *VIM* in hepatocellular carcinoma (34), and squamous cell carcinoma CSCs (35).

Interestingly, the expression of miR-15b washigher in patients’ serum and melanospheres than in the control group. However, in patient samples, its expression in melanoma biopsies was lower than normal tissues. This difference in the pattern of expression may be associated with recurrent-free survivalin patients (36).

A combination of ingenuity analysis for potential regulators and target genes and examination of biological pathways targeted by the deregulated miRNAs, indicated that the regulatory network around miR-205, -9, -203, and -15b was most prominent in our data. The significant association of combined expression of these miRNAs with overall survival of melanoma patients was confirmed through the TCGA data. Among all target genes, negative correlation between miR-205, -203 and -15b expression and *VEGF* was observed in melanospheres. Also, our results provide evidence for negative correlation between these miRNAs and *TNF* by miR-205, -203 and -15b in tumors. Whereas, miR-9 shows a positive regulatory effect on *TNF* expression in D10 and A375 melanospheres and melanoma patient samples. In fact, in patients’ samples, *TNF *expression was increased concomitant with a high expression of miR-9 and low expression of miR-203. This data was verified by the expression patterns in melanospheres, in which reduced *TNF* expression coincided with higher expression of miR-203 (D10- and A375-melanospheres). These data can confirm the important role of miR-203 in regulating the expression of the pro-inflammatory cytokine TNF. Importantly, treatment of melanoma cells with TNF suppresses CSCs differentiation through PI3K/AKT signaling (37). However, the role of TNF as an intrinsic factor in melanoma stem cell fate requires further studies. VEGF is another factor that can affect the growth and metastasis of melanoma (38). We suggested here that miR-205, -203, and -15b could negatively regulate VEGF expression. Although we could not find an explanation for the observed lowered expression of *VEGF* in melanospheres, but it paly important role in the VEGF-CSC axis in a variety of tumors, including melanoma (39).

## Conclusion

Based on our findings, miR-205, -15b, -203, -9 were selected as the key miRNAs linked to tumor/normal status, which can regulate the pluripotency, proliferation, and motility of malignant cells. However, further studies are required to find the exact mechanisms underlying the combinatory effects of the abovementioned miRNAs.

## Supplementary PDF


