# Supplementary material for: An Integrative Analysis of The Micro-RNAs Contributing in Stemness, Metastasis and B-Raf Pathways in Malignant Melanoma and Melanoma Stem Cell
Source: Cell J. 2021 Jul 17;23(3):261–72. doi: 10.22074/cellj.2021.7311 (PMC8286452; doi:10.22074/cellj.2021.7311)
Supplement: Supplementary file 1 [file Cell-J-23-261-s01.pdf]

CELL JOURNAL (Yakhteh)

Supplementary Information for

An Integrative Analysis of The Micro-RNAs Contributing in Stemness, Metastasis and B-Raf Pathways in Malignant Melanoma and Melanoma Stem Cell

Parisa Sahranavardfard, Ph.D.[1], Zahra Madjd, M.D., Ph.D.[2], Amirnader Emami Razavi, M.D.[3], Alireza Ghanadan, M.D.[3, 4], Javad Firouzi, M.Sc.[1], Pardis Khosravani, M.Sc.[1], Saeid Ghavami, Ph.D.[5, 6, 7, 8]*, Esmaeil Ebrahimie, Ph.D.[9, 10]*, Marzieh Ebrahimi, Ph.D.[1]*

1. Department of Stem Cells and Developmental Biology, Cell Science Research Center, Royan Institute for Stem Cell Biology and Technology, ACECR, Tehran, Iran
2. Department of Pathology, Iran University of Medical Sciences, Tehran, Iran
3. Iran National Tumor Bank, Cancer Institute of Iran, Tehran University of Medical Sciences, Tehran, Iran
4. Department of Dermatopathology, Razi Skin Hospital, Tehran University of Medical Sciences, Tehran, Iran
5. Department of Human Anatomy and Cell Sciences, University of Manitoba, Manitoba, Canada
6. Biology of Breathing, Children Hospital Research Institute of Manitoba, University of Manitoba, Winnipeg, Canada
7. Autophagy Research Center, Shiraz University of Medical Sciences, Shiraz, Iran
8. Research Institute in Oncology and Hematology, Cancer Care Manitoba, University of Manitoba, Winnipeg, Canada
9. School of Animal and Veterinary Sciences, The University of Adelaide, Adelaide, Australia
10. Genomics Research Platform, School of Life Sciences, College of Science, Health and Engineering, La Trobe University, Melbourne, Australia

**Corresponding Addresses: Department of Human Anatomy and Cell Sciences, University of Manitoba, Manitoba, Canada*
School of Animal and Veterinary Sciences, The University of Adelaide, Adelaide, Australia
Department of Stem Cells and Developmental Biology, Cell Science Research Center, Royan Institute for Stem Cell Biology and Technology, ACECR, Tehran, Iran
Emails: saeid.ghavami@umanitoba.ca, esmaeil.ebrahimie@adelaide.edu.au, mebrahimi@royaninstitute.org

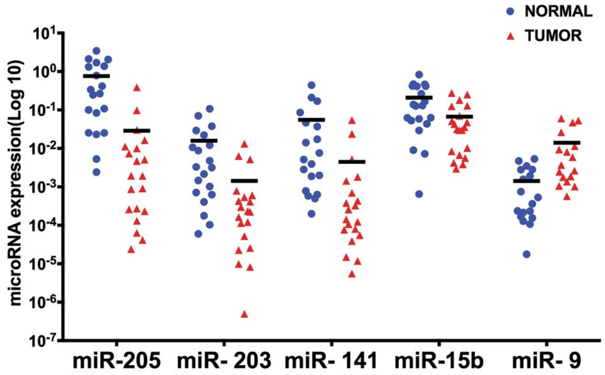

	miR-205	miR- 203	miR- 141	miR-15b	miR- 9
SNAIL	-	+	-	-	+
ZEB	+	+	+	-	-
CDH1	-	-	-	-	+
SMAD2/3	-	+	-	+	-
CCND1	-	-	-	+	+
SOX2	-	-	-	-	+
POU5F1	-	-	+	-	-
BRAF	-	-	-	-	+
ERK	-	+	-	+	-
MEK	-	-	-	-	+
CDK4	-	-	-	+	-

Fig.S1: Expression pattern of differentially miRNAs expressed with direct validated targets. According to miRTarBase 6.0 validated data, miR-205, -203, -141, -15b, -9 had at least one target in the EMT pathway: miR-205 and miR-141 target *ZEB*; miR-203 targets *ZEB*, *SNAIL,* and *SMAD2*; miR-15b targets *SMAD2*; miR-9 targets *CDH1* and *SNAIL*. Also, miR-141 inhibits *POU5F1* and miR-9 directly targets *SOX2* as stemness genes. In addition, miR-9, -15b, and -203 are involved in the BRAF pathway by directly targeting *BRAF* or one of its downstream factors, like *ERK*, *MEK* or *CCND1*.

Table S1: Different algorithms of attribute weightings

Number	Algorithm model	Definition
1.	principle component analysis (PCA)	Taking the coefficients of the first component of PCA as feature weights
2.	Deviation	Creating weights from the standard deviations of all attributes
3.	Relief	Measuring the relevance of features by sampling examples and comparing the value of the current feature for the nearest example of the same and of a different class
4.	Support vector machine (SVM)	Taking the coefficients of the normal vector as weight of features
5.	Gini index	Calculating the relevance of an attribute by computing the Gini index of the class distribution
6.	Rule	Calculating the relevance of an attribute by computing the error rate of a OneR Model on the example set without this feature
7.	Chi Squared statistic	Calculating the relevance of an attribute by computing the value of the chi-squared statistic with respect to the class attribute
8.	Information gain	Calculating the relevance of an attribute by computing the information gain in class distribution
9.	Uncertainty	Calculating the relevance of an attribute by measuring<br><br>the symmetrical uncertainty with respect to the class
10.	Information gain ratio	Calculating the relevance of an attribute by computing the information gain ratio for the class distribution

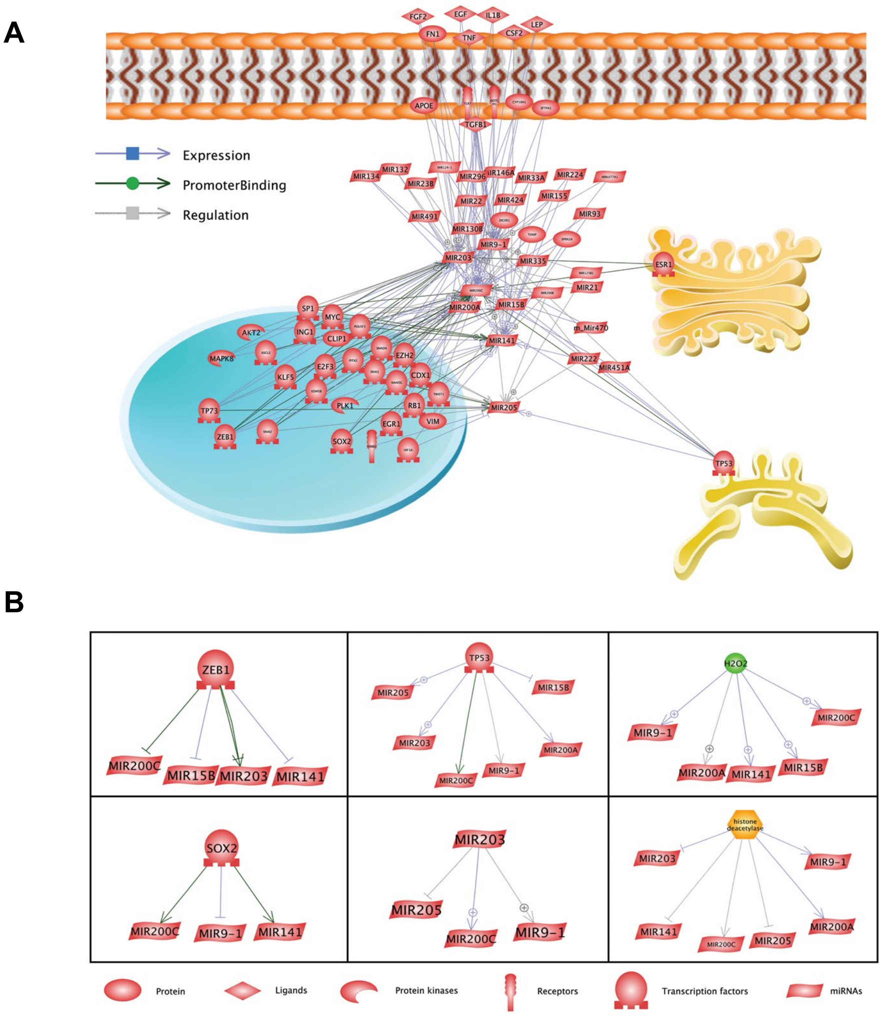

Fig.S2: Regulatory network analysis to find more effective miRNAs in melanoma progression. **A.** Based on regulator discovery, the selected miRNAs were modulated by most intracellular components, including the nucleus, Golgi apparatus, endoplasmic reticulum, and cell membrane and **B.** Main significant sub-networks enriched by differentially expressed miRNAs of melanoma and normal tissues. The common regulators were TP53 and histone deacetylase that regulated six of the seven selected miRNAs.

Table S2: Melanoma sample characterization

Characteristic	Value
Age (Y, mean age ± standard deviation)	59 ± 13.89
Gender (%)	
Female	25
Male	75
Tumor size (%)	
≤4.1 mm	67
>4.1 mm	33
Metastasis (%)	
Yes	92
No	8
Dermal invasion (%)	
Yes	67
No	33%
Local recurrence (%)	
Yes	42
No	33
No data	25
Tumor-infiltrating lymphocytes (%)	
Absent	84
Moderate	8
Brisk	8
Margin involvement (%)	
Yes	17
No	83
Breslow thickness (%)	
Thin (≤1 mm)	67
Thick (>1 mm)	33
Lymph node involvement (%)	
Yes	67
No	33

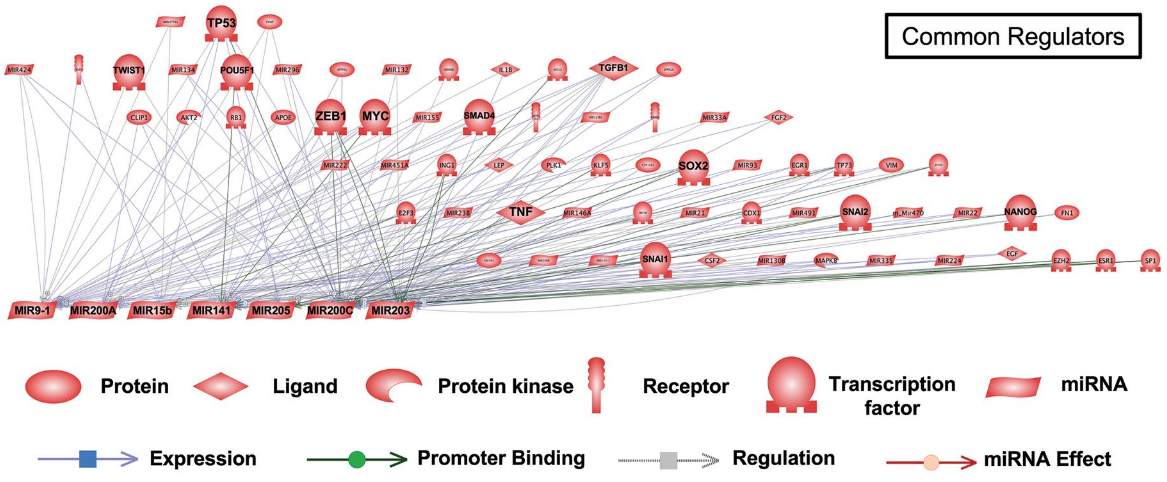

Fig.S3: Regulator discovery for differentially expressed miRNAs. Analysis of common regulator showed SOX2 and some of the main EMT markers, such as SNAI1, SNAI2, ZEB1, and TGFB1, as common regulators of significant miRNAs.

Table S3: Melanoma sample characterization

Characteristic	Value		
Age (Y, mean age ± standard deviation)	65 ± 12.99		
Gender (%)			
Female	55		
Male	45		
Ethnicity (%)			
Persian	30		
Azari	45		
Gilaki	15		
Lur	5		
Unknown	5		
Tumor size			
Tumor diameter (mm, mean ± SD)	30.61 ± 18.47		
Tumor depth (mm, mean ± SD)	14.41 ± 12.55		
Site of primary (%)			
Upper limb and shoulder	5		
Scalp and neck	15		
Lower limb and hip	45		
Other and unspecified parts of body	35		
Extent of disease (%)	Yes	No	Unknown
Bone invasion	5	65	25
Perineural invasion	10	65	30
Dermal lymph invasion	10	65	30
Vascular invasion	10	65	30
Grade (%)			
I	5		
II	30		
III	20		
IV	45		
Unknown			
Stage (%)			
II	65		
III/IV	35		
Donor status			
Alive	50		
DECEASED	50		

A

TCGA Skin Cutaneous Melanoma [SKCM] DATA SURVIVAL MEASURE –OVERAL SURVIVAL

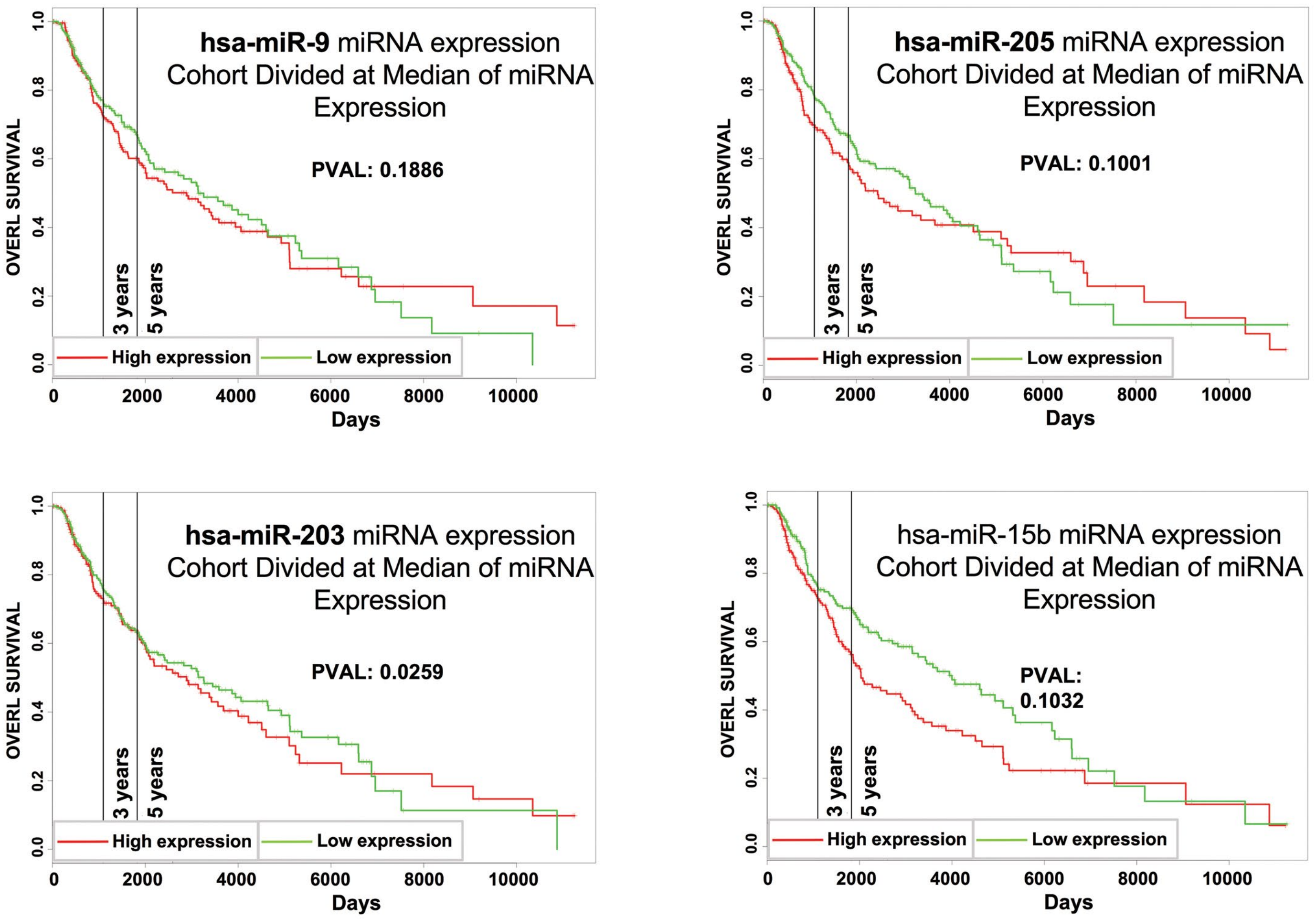

B

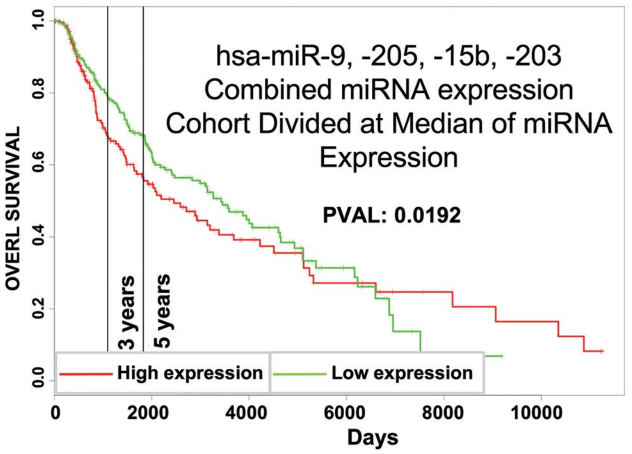

Fig.S4: The overall survival rate associated with miR-203, -205, -15b, and -9. **A.** The individual expression levels of miR-9 (P=0.1886), -205 (P=0.1001), and -15b (P=0.1032) were not associated with reduced overall survival of melanoma patients. **B.** The simultaneously expression of miR-203, -205, -15b, and -9 (P=0.0192). However, miR-203 (P=0.0259) alone and the simultaneously expression of miR-203, -205, -15b, and -9 (P=0.0192) showed a significant association with reduced overall survival of patients. The PROGmiR tool was used for analysis of the melanoma expression data from the TCGA dataset, including 163 cases of skin cutaneous melanoma.

Table S4: Clinical and Laboratory characteristics of patient samples

Sample Code	Age (Y)	Gender	Ethnicity	Site of primary	Ulceration	Tumor size (mm, Diameter/ Depth)	Extent of disease	Pathological T	Pathological N	Clinical M	Grade	Donor statue
A00017	68	M	Persian	Lower limb and hip	Yes	(15/2)	No	T2b	NX	MX	X	DECEASED
A00042	76	M	Persian	Unspecified parts of body	Yes	(35/35)	No	T3	NX	MX	IV	ALIVE
A00186	52	F	Gilaki	Unspecified parts of body	Yes	(30/8)	No	T4a	NX	MX	III	ALIVE
A01650	55	F	Unknown	Lower limb and hip	No	(35/35)	Unknown	T4a	NX	M0	III	ALIVE
A01679	38	F	Azari	Lower limb and hip	Yes	(55/25)	No	T4b	N0	M0	X	ALIVE
A01732	63	M	Azari	Scalp and neck	No	(40/35)	No	T4a	NX	M0	III	ALIVE
A00151	62	F	Azari	Lower limb and hip	Yes	(10/5)	Dermal lymph and vascular invasion	T4b	NX	MX	II	ALIVE
A00320	74	F	Gilaki	Scalp and neck	No	(9/4)	Perineural invasion	T3a	NX	M0	Null	ALIVE
A00678	82	M	Azari	Unspecified parts of body	Unknown	(80/-)	Unknown	T4a	N2b	M1a	IV	DECEASED
A00856	48	F	Azari	Upper limb and shoulder	No	(15/-)	Unknown	TX	N0	M0	X	ALIVE
A01185	47	M	Azari	Lower limb and hip	Yes	(20/3)	No	T3b	N3	M0	IV	ALIVE
A01358	83	F	Gilaki	Unspecified parts of body	Yes	(25/6)	No	T4b	N0	MX	III	DECEASED
A01371	80	F	Azari	Lower limb and hip	Yes	(55/3)	Unknown	T3b	N0	M0	III	DECEASED
A00786	82	F	Persian	Unspecified parts of body	No	(10/9)	Perineural invasion	T4a	N0	MX	X	DECEASED
A01192	52	F	Azari	Lower limb and hip	No	(-/13)	Unknown	T4a	N3	MX	IV	DECEASED
A00743	65	M	Persian	Unspecified parts of body	Yes	(-/-)	Bone invasion	TX	N2	MX	X	DECEASED
A01483	65	M	Persian	Lower limb and hip	No	(30/30)	Dermal lymph and vascular invasion	T4	NX	MX	X	DECEASED
A01556	70	M	Azari	Unspecified parts of body	Yes	(30/10)	No	T2	NX	MX	Null	ALIVE
A00871	66	F	Lur	Lower limb and hip	No	(35/18)	No	T4a	NX	MX	III	DECEASED
A00278	72	M	Persian	Scalp and neck	Null	(22/4)	Unknown	T3a	N2b	MX	Null	DECEASED

F; Female, M; Male, T; Tumor, N; Node, and M; Metastasis.

Table S5: Attribute weighting models select the most important microRNAs linked to tumor/normal status, based on 10 different statistical approaches

Weight_ PCA	Weight_ SVM	Weight_ Relief	Weight_ Uncertainty	Weight_ Gini Index	Weight_Chi Squared	Weight_ Deviation	Weight_ Rule	Weight_Info Gain Ratio	Weight_ Info Gain	Attribute	Number of attribute weighting models selected the microRNAs as important (cut off 0.9)
0.0	0.2	0.2	0.9	1.0	0.4	0.0	0.4	1.0	1.0	miR-205	4
0.0	1.0	1.0	1.0	0.3	1.0	0.0	0.5	0.4	0.3	miR-15b	4
1.0	0.4	0.0	0.1	0.2	0.0	1.0	0.9	0.0	0.2	miR-221	3

PCA; Principal component analysis and SVMs; Support vector machines.
